# Generation of Functional CLL-Specific Cord Blood CTL Using CD40-Ligated CLL APC

**DOI:** 10.1371/journal.pone.0051390

**Published:** 2012-12-19

**Authors:** William K. Decker, Nina Shah, Dongxia Xing, Ruth Lapushin, Sufang Li, Simon N. Robinson, Hong Yang, Simrit Parmar, Matthew M. Halpert, Michael J. Keating, John G. Gribben, Jeffrey J. Molldrem, Elizabeth J. Shpall, William G. Wierda

**Affiliations:** 1 Baylor College of Medicine, Department of Pathology and Immunology, Houston, Texas, United States of America; 2 Department of Stem Cell Transplantation and Cellular Therapy, The University of Texas MD Anderson Cancer Center, Houston, Texas, United States of America; 3 Department of Leukemia, The University of Texas MD Anderson Cancer Center, Houston, Texas, United States of America; 4 Institute of Cancer, Barts and The London School of Medicine, Charterhouse Square, London, United Kingdom; Mie University Graduate School of Medicine, Japan

## Abstract

Though remissions have been observed following allo-HSCT for the treatment of CLL, many CLL patients are ineligible for transplant due to the lack of HLA-compatible donors. The use of umbilical cord blood (UCB) permits transplantation of many patients who lack HLA-compatible donors due to reduced requirements for stringent HLA matching between graft and recipient; however, disease relapse remains a concern with this modality. The generation of CLL-specific CTL from UCB T-cells, primed and expanded against the leukemic clone, might enhance the GVL effect and improve outcomes with UCB transplantation. Here we report the generation of functional, CLL-specific CTL using CD40-ligated CLL cells to prime partially-HLA matched UCB T-cells. Functionality and specificity were demonstrated by immune synapse assay, IFN-γ ELISpot, multi-parametric intracellular cytokine flow cytometry, and ^51^Cr release assay. The use of patient-specific, non-CLL controls demonstrated the generation of both alloantigen and CLL-specific responses. Subsequently, we developed a clinically-applicable procedure permitting separation of alloreactive CTL from leukemia-specific CTL. Leukemia-specific CTL were able to mediate *in vivo* killing of CLL in humanized mice without concurrent or subsequent development of xenoGVHD. Our results demonstrate that generation of CLL-specific effectors from UCB is feasible and practical, and the results support further exploration of this strategy as a treatment modality for CLL.

## Introduction

B-cell chronic lymphocytic leukemia (CLL) is the most prevalent of the adult leukemias, accounting for at least 180,000 new diagnoses worldwide every year [1]. While CLL is effectively treated with a variety of chemotherapeutic or antibody-based regimens, it is a chronic disease and cure is not achieved with conventional chemotherapy [2]. There is clear evidence that a graft versus leukemia (GVL) effect exists in CLL [3]; [4], and a number of studies have successfully demonstrated durable remissions following allogeneic hematopoietic stem cell transplantation (allo-HSCT) for poor-risk, relapsed, or refractory CLL [5]; [6]. Umbilical cord blood (UCB) is being used with increasing frequency as a stem cell source for allotransplant among patients lacking HLA-compatible donors. In spite of successful outcomes following allo-HSCT, disease relapse remains a significant contributor to post-transplant mortality in patients with CLL. Accordingly, a number of strategies have been employed in an effort to amplify and perpetuate GVL in conjunction with allo-HSCT including the use of rituximab [7], non-myeloablative reduced-intensity conditioning regimens, and donor lymphocyte infusion (DLI) [4]–[8].

Consistent with a GVL affect, CLL cells are known to express a variety of tumor-associated antigens (TAA) that distinguish CLL from non-neoplastic cell types. Patient-specific idiotype determinants, fibromodulin, MDM2, survivin, and KW-13 are some of the CLL-specific antigens against which cellular immune responses are known to be directed [9]–[13], and a variety of different strategies have been employed to exploit the existence of a disease-specific antigenic profile [14]–[16]. One promising approach attempted clinically involved vaccination of patients with autologous CLL cells engineered to express CD154 (CD40L) [17]. The engagement of CD40 by its ligand on the surface of CLL cells upregulates many costimulatory molecules, thereby transforming the CLL cells into antigen-presenting cells (APCs) capable of stimulating CLL-specific T-cell responses [17]–[19]. Though this strategy produced detectable clinical responses, such responses were typically short-lived. The transient nature of a T-cell approach was not wholly unexpected given the well-documented ability of CLL to suppress T-cell function [20]–[22]. Recent work by our group has demonstrated that umbilical cord blood (UCB) T-cells may have some capacity to overcome immunosuppressive affects typically exerted by CLL [23]. While the precise mechanism of this phenomenon is not completely understood, these observations led to the hypothesis that partially-HLA matched UCB T-cells might be able to mediate specific recognition and killing of CLL. The generation of such CLL-specific T-cells from cord blood could be of significant clinical benefit either in the setting of UCB transplantation or as a third party T cell therapy after matched-related or matched-unrelated donor HSCT. While far greater experience exists with traditional stem cell sources for patients with CLL, there is mounting evidence that UCB transplant for CLL is feasible and comparable to traditional graft sources, even with reduced-intensity conditioning regimens [24]; [25].

## Materials and Methods

### Ethics statement

All animal work was performed under an MDACC IRB approved animal protocol. All mice were euthanized under anesthesia by cervical dislocation and experienced no pain or suffering. All patient samples were made available for research use under an MDACC IRB approved clinical protocol with informed, written consent of the patient. All umbilical cord blood samples were made available for research use under MDACC IRB approved protocols LAB02-630 and LAB03-796 with informed, written consent of the mother.

### Generation of CLL-APC

CLL APCs were generated from peripheral blood lymphocytes of CLL patient samples donated under an IRB-approved protocol. CLL peripheral blood mononuclear cell (PBMC) samples used in these experiments consisted of 80–99% CLL cells as determined by CLIA-compliant clinical flow cytometry. Patient PBMCs were separated from red cells on a Ficoll gradient and cryopreserved. Upon thawing, patient PBMCs were resuspended at 2×10^7^/ml in AIM-V medium (Invitrogen, Carlsbad, CA). Cells were then transduced with an adenoviral construct encoding murine CD40L (mCD154, surface stable on human cells) [17] by a 1∶1 incubation of cells and virus at 37°C for 48 hours. Transduction and subsequent ligation of CD40 was verified by monitoring expression of mCD154 and upregulation of CD95 and/or CD80 by flow cytometry. Transduced CLL-APC were γ-irradiated at 3,000 cGy.

### Priming and Expansion of Partially-HLA matched UCB T-cells

Partially HLA matched (at least 4/6 of HLA-A, B, and DR loci) non-adherent UCB lymphocytes were incubated 5∶1 with CLL-APC in RPMI-1640 (Invitrogen) supplemented with 10% human AB serum (Gemini Bioproducts, West Sacramento, CA), 2 mM l-glutamine (Invitrogen), and 1% Antibiotic/Antimycotic (Invitrogen). After four days, cultures were supplemented with 100 U/ml IL-2 (Chiron, Emeryville, CA), 10 ng/ml IL-7 (R&D Systems, Minneapolis, MN), and 5 ng/ml IL-15 (R&D Systems), and resupplemented with fresh cytokines every 2 days thereafter. IL-7 and IL-15 were only supplied during the first 10 days of expansion. Expanding UCB lymphocytes were restimulated with cryopreserved CLL-APC 7–10 days after the primary stimulation and another 7–10 after the secondary stimulation. Expanded UCB cells were generated against 14 different combinations of UCB units and CLL patient samples.

### Immunoassays

IFN-γ ELISpot and ^51^Cr release assays were performed as previously described [26] using non-CD40 ligated CLL cells as targets, expanded CD8^+^ cells isolated from the same CLL patient sample (alloantigen control targets), and K562 cells (NK cell activity controls). E∶T ratios are reported at 20∶1 unless otherwise indicated. CD8^+^ cells were obtained by isolation and expansion of patient CD3^+^ with anti-CD3/anti-CD28 beads (Invitrogen) followed by depletion of CD4^+^ cells with magnetic beads (Miltenyi-Biotec, Auburn, CA). Expansion of CD8^+^ cells with anti-CD3/anti-CD28 beads generates CD8^+^ cells with an activated phenotype. In HLA blocking experiments, MHC class I-mediated lysis was blocked using the W6/32 pan-class I blocking antibody (Dako, Carpinteria, CA) at a dilution of 50∶1 (3.5 µg/ml). Immune synapse assay was performed as previously described [27] using unmanipulated CLL cells as targets. Multiparametric intracellular cytokine and surface marker flow cytometry assays were performed as previously described [26]. TCR Vβ spectratyping was performed as previously described [28].

### *In Vitro* Separation of GVH from GVL

Bulk T-cell products stimulated twice previously with CLL-APC were cultured with γ-irradiated, bead-expanded CD8^+^ cells derived from the same CLL patient as were the APC. After 24 hours co-culture, the T-cell product was CD25-depleted by magnetic separation using either a research-grade strategy (anti-CD25/biotin primary [Biolegend, San Diego, CA] followed by anti-biotin microbeads [Miltenyi-Biotec]) or a GMP-compliant strategy (anti-CD25 microbeads [Miltenyi-Biotec]). Subsequently, the CD25-negative fraction was stimulated a third time with CLL-APC and subsequently characterized by immunoassay.

### *In Vivo* Modeling

Humanized NOD/scid/IL-2Rγ^−/−^ (NSG) mice were engrafted with CLL as described previously [29]. Briefly, NSG mice were irradiated at 320 cGy after which they were humanized by i.v. administration of 1.5×10^5^ CD34-selected umbilical cord blood cells. Mice were bled every two weeks and analyzed by flow cytometry to monitor for the emergence of human CD3^+^ T-cells. Upon detection of T-cells, mice were engrafted with 5×10^6^ CFSE-labeled CLL cells. Engrafted CLL was derived from the same clone as was used to generate CLL-APC for expansion of UCB-CTL. Allogeneic interaction between UCB-derived T-cells and the few T-cells that accompany the CLL graft is hypothesized to produce the cytokines necessary for CLL engraftment [26]. Following verification of CLL engraftment by CFSE dilution (1–2 weeks post-administration of the CLL graft), engrafted mice received a single injection of 5×10^6^ CLL-specific CTL i.v. B-cell content of engrafted mice was monitored over the next six weeks by flow analysis of blood, spleen, and marrow by staining with anti-CD5, CD19, CD23, and CD45.

### Statistical Analysis

Statistical differences were calculated by Student's unpaired two-tailed t-test unless stated otherwise. Significance was defined as p≤0.05. Error bars in all figures = +/−SD. All experimental observations were derived from at least nine independent experiments unless stated otherwise.

## Results

### Generation of CLL APC and characterization of partially HLA-matched UCB lymphocytes

**Figure 1:**
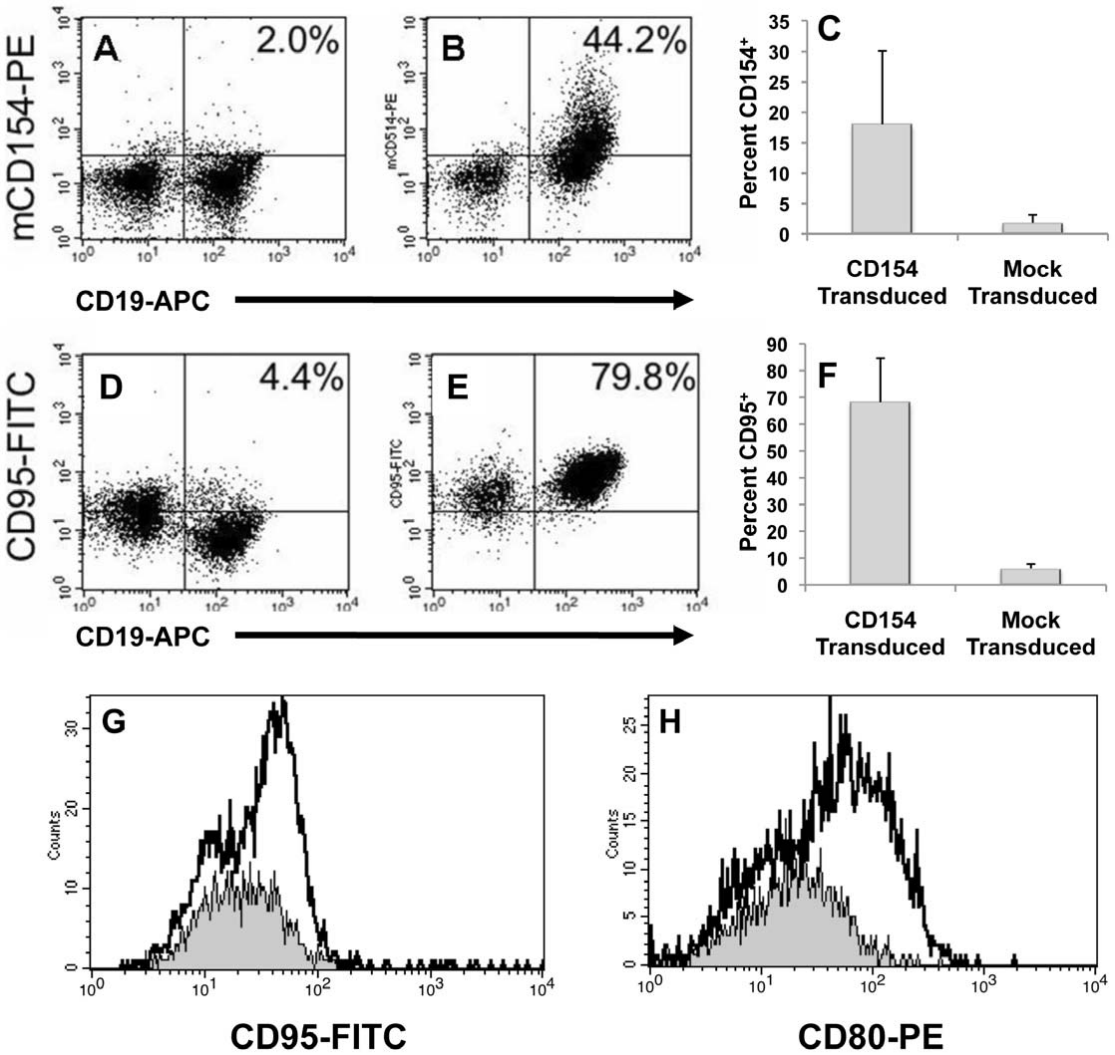
Characterization of CLL-APC. Mock-treated (A) or vector-treated (B) CLL cells indicate successful transduction of CLL cells with surface stable mCD154 (mCD40L) leading to upregulation of CD95 expression on (E) vector-treated but not (D) mock-treated CLL cells. Figure 1C graphically represents CD154 transduction efficiency of 12 independent experiments and Figure 1F graphically represents post-transduction CD95 upregulation of those same experiments. Error Bars = +/−SD. Upregulation of CD95 (G) was shown to correlate with upregulation of costimulatory marker CD80 (H) following treatment with CD40L-expressing vector (unshaded area). Gray shaded area indicates cells treated with control viral vector.

To generate CLL-APC, patient PBMCs were transduced with an adenoviral vector encoding murine CD154 (surface stable on human B-cells) [17]. Figure 1A and B demonstrate that CD19^+^ CLL cells could be successfully transduced as demonstrated by flow staining with anti-mouse CD154 (panels A and B). Transduction efficiency of CLL cells was variable but generally robust ***(average transduction efficiency = 18.0%, range = 2.6%–44.3%, n = 12;*** ***Figure 1C*** ***)***; however, even at low transduction efficiencies, subsequent upregulation of CD95 was generally good due to bystander effects. Stimulation of CD40 and modulation of CLL cell phenotype were determined by flow staining with anti-CD95 (Figures 1D, 1E, and 1F). We demonstrated that upregulation of CD95 expression was well-correlated with concomitant upregulation of traditional costimulatory molecules like CD80 (Figures 1G and 1H) as reported previously [17]. Transduced CLL cells were compared to both mock-transduced cells (PBS) and cells transduced with an irrelevant adenovector, neither of which demonstrated appreciable upregulation of CD95 or CD80 from baseline. In 11 independent experiments, costimulatory marker expression following CD40 ligation was at least 0.5× log-fold higher than unligated controls.

**Figure 2:**
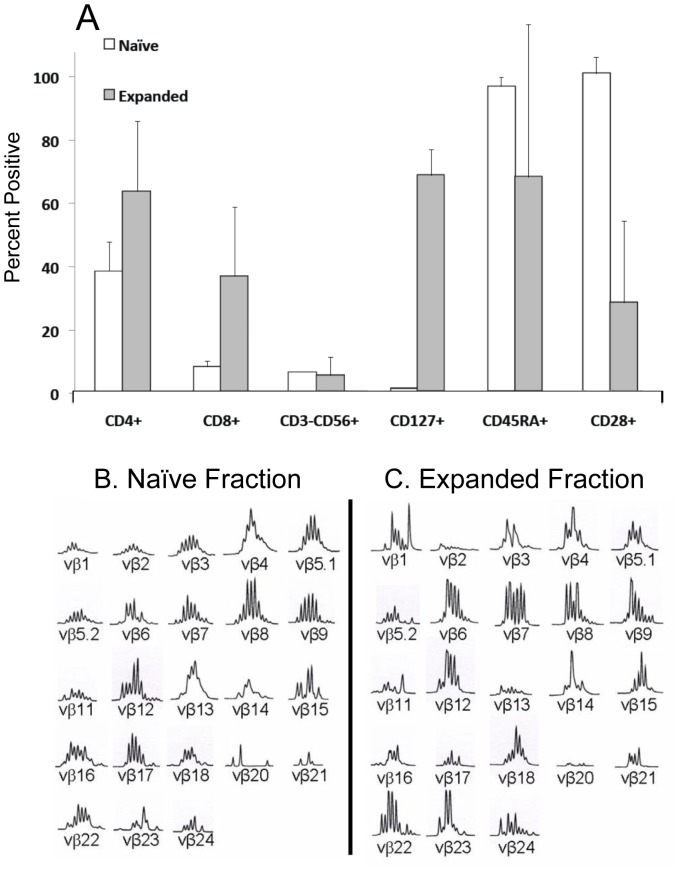
Characterization of UCB T-cells expanded by CLL-APC. A. UCB cell products expanded by CLL-APC consisted almost entirely of CD3^+^ cells with very few contaminating CD3^−^CD56^+^ cells. CD3^+^ cells upregulated CD127 from undetectable levels pre-stimulation, indicating a memory phenotype. Error Bars = +/−SD. CD45RA and CD28 were also significantly downregulated among expanded T-cells. Following three stimulations with CLL-APC, expanded UCB lymphocytes were spectratyped to determine Vβ repertoire polyclonality in comparison to the naïve, unstimulated fraction. B. Vβ locus spectratype distribution of naïve, unstimulated lymphocytes. C. Vβ locus spectratype distribution of stimulated, expanded lymphocytes. One of seven experiments shown. Note that repertoire polyclonality is maintained.

Partially-HLA matched UCB lymphocytes (typically 4/6) were expanded by three successive stimulations with CLL-APC. ***Table 1*** ***demonstrates the expansion kinetics of nine different UCB T-cell populations. In aggregate, lymphocytes underwent a 5.7+/−5.5 fold expansion over a period of 21 days***. After three stimulations with CLL-APC, the product consisted almost entirely of CD3^+^ cells with very few CD3^−^CD56^+^ NK cells present (Figure 2A). A majority of expanded UCB T-cells demonstrated an upregulation of CD127 ***(69.3%, range 64.9%–76.6%)*** from undetectable pre-expansion levels (Figure 2A), indicating a memory phenotype. ***CD3^+^ cells averaged 36% CD8^+^ content (range 11%–53%,*** ***Figure 2A*** ***)*** **.** CD19^+^ cells were not detected in any proliferating cell population (data not shown).

**Table 1:** Umbilical Cord Blood Lymphocyte Expansion Properties.

Patient	Cord Unit	HLA Matches	Starting (×10^6^ cells)	Actual Max (×10^6^ cells)	Calculated Max (×10^6^ cells)*
1	003969	4/6	10	26	208
2	003859	4/6	10	32	256
3	004442	4/6	9	23	185
4	000741	4/6	10	46	364
4	003187	4/6	10	41	326
5	010566	4/6 (6/8)	43	135	268
6	008827	4/6 (6/8)	32	74	149
8	006481	5/6 (7/8)	44	876	2,110
9	009139	5/6 (6/8)	83	850	901

* Calculated Max extrapolates the number of cells that would have been available had an entire 20% cord fraction been used in the expansion.

Three different expanded UCB T-cell lines were spectratyped to assess clonality, given that a tumor might avoid a monoclonal or oligoclonal response by selective mutation of a key antigen. Figure 2B and 2C show a representative example of a full spectratype analysis of the naïve, unstimulated UCB lymphocyte fraction (2B) and the same fraction following three stimulations with CLL-APC (2C). For any Vβ locus, polyclonality is indicated by a Poisson distribution of spectral peaks. The stimulated repertoire appeared to remain highly polyclonal, supporting a hypothesis of retained T-cell specificity against multiple antigens.

**Figure 3:**
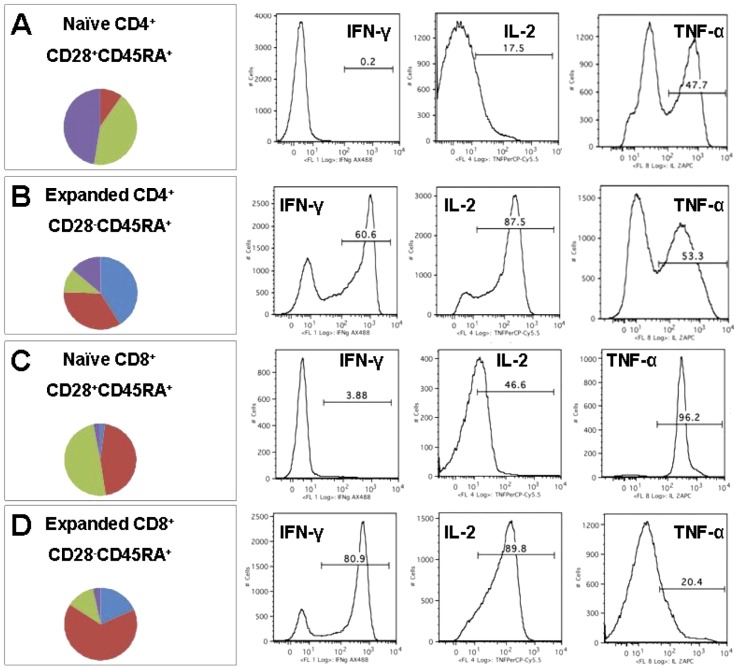
UCB T-cells stimulated by partially HLA-matched CLL-APC exhibit functional cytokine profiles and variegated memory phenotypes. Fractions of expanded and naïve T-cells were treated with PMA/ionomycin and then analyzed by seven color flow cytometry for CD28/CD45RA memory marker expression and Th-1 cytokine expression profile (IFN-γ, IL-2, and TNF-α). A. Naïve CD4^+^ cell fraction. B. Naïve CD8^+^ cell fraction. C. Expanded CD4^+^ cell fraction. D. Expanded CD8^+^ cell fraction. Naïve cells were almost exclusively CD28^+^CD45RA^+^ whereas expanded cells were predominantly CD28^−^CD45RA^+^. A sizeable minority of CD4^+^ cells retained CD28 surface expression. Blue = fraction of cells positive for all three cytokines analyzed. Red = fraction of cells positive for two of three cytokines analyzed. Green = fraction of cells positive for one of three cytokines analyzed. Purple = fraction of cells negative for all three cytokines analyzed.

Expanded CLL-specific T-cells and the naïve UCB fractions from which they were derived were both treated with PMA/ionomycin and analyzed by multi-parametric flow cytometry to determine cytokine secretion profile and memory phenotype as indicated by staining with CD28 and CD45RA. Nearly all cells derived from naïve fractions were CD28^+^CD45RA^+^ (Table 2). Naïve cells did not produce any IFN-γ and only low levels of IL-2. Interestingly, half of naïve UCB CD4^+^ and most naïve UCB CD8^+^ cells generated high levels of TNF-α (Figures 3A, 3C, and Table 2). In contrast, a plurality of stimulated CLL-specific UCB T-cells were CD28^−^CD45RA^+^ (Figures 3C, 3D, and Table 2). The number of CD28^−^CD45RA^+^ cells was well-correlated with the number of CD127^+^ cells. Most of the stimulated cells produced IFN-γ and high levels of IL-2. As with the naïve population, about half of the stimulated CD4^+^ cells produced TNF-α; however, most of the stimulated CD8^+^ cells no longer generated TNF-α (3B and 3D). In each panel of Figure 3, the plots shown are representative of five independent experiments. A summation of the data is found in Table 2.

**Table 2:** Umbilical Cord Blood Lymphocyte Memory Phenotypes.

Phenotype	CD4^+^		CD8^+^	
	Naïve	Expanded	Naïve	Expanded
CD28^+^CD45RA^−^	4.5+/−3.0%	9.4+/−11.6%	0.8+/−1.1%	5.0+/−7.2%
CD28^+^CD45RA^+^	94.9+/−3.1%	24.9+/−20.3%	98.4+/−1.7%	16.0+/−11.8%
CD28^−^CD45RA^+^	0.1+/−0.1%	36.5+/−29.7%	0.5+/−0.5%	59.0+/−35.5%
CD28^−^CD45RA^−^	0.5+/−0.3%	29.2+/−33.4%	0.3+/−0.4%	20.1+/−27.4%

### UCB T-cells stimulated by partially HLA-matched CLL-APC form functional immunological synapses and preferentially secrete IFN-γ when complexed with unmanipulated CLL cells

**Figure 4:**
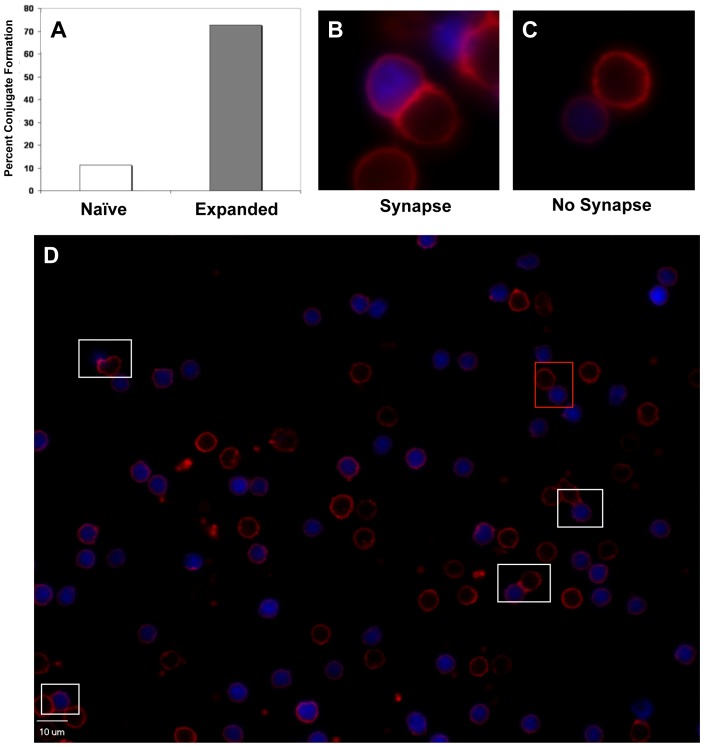
UCB-derived CLL-specific T-cells form immunosynaptic complexes with CLL target cells. (A) UCB-derived CLL-specific T-cells formed immunosynaptic complexes in 72% of conjugation events with CLL cells while naïve T-cells could only form such complexes in 11% of conjugations. Examples of synaptic complex (B) vs. non-synaptic complex (C) are shown. (D) indicates a typical analytic field in which synaptic complexes are identified by white boxes and non-synaptic complexes by red boxes. In practice, at least 20 such field were evaluated for both naïve and expanded lymphocyte populations.

CLL cells can be profoundly immunosuppressive [20]–[22], a characteristic that has hampered previous attempts at adoptive immunotherapy. In order to verify, as previously reported [23], that UCB-derived CLL-primed T-cells retained immune functionality and/or specificity for unmodified CLL targets, we performed an immunosynapse assay as well as IFN-γ ELISpot assays. The formation of the immune synapse is the first step by which targets are identified and eradicated, and previous work has demonstrated that CLL cells can suppress the formation of immune synapses when complexed with healthy allogeneic T-cells [20]. As demonstrated by the experiment shown (Figure 4A), 72% of UCB T-cells primed by CD40-ligated CLL APC retained the ability to form f-actin immunoconjugates with unligated CLL cells. In comparison, only 11% of naïve T-cells from the same UCB graft had the ability to form synapses with CLL cells derived from the same donor. Figures 4B and 4C indicate a typical immunoconjugate between a blue-labeled CLL cell and B) a CLL-specific UCB T-cell vs. C) a naïve UCB T-cell. A representative field of CLL-specific UCB T-cells is shown in Figure 4D with immunosynaptic conjugates boxed in white and nonproductive conjugates boxed in red. In this representative field, 4/5 conjugates or 80% form a polarized synapse.

**Figure 5:**
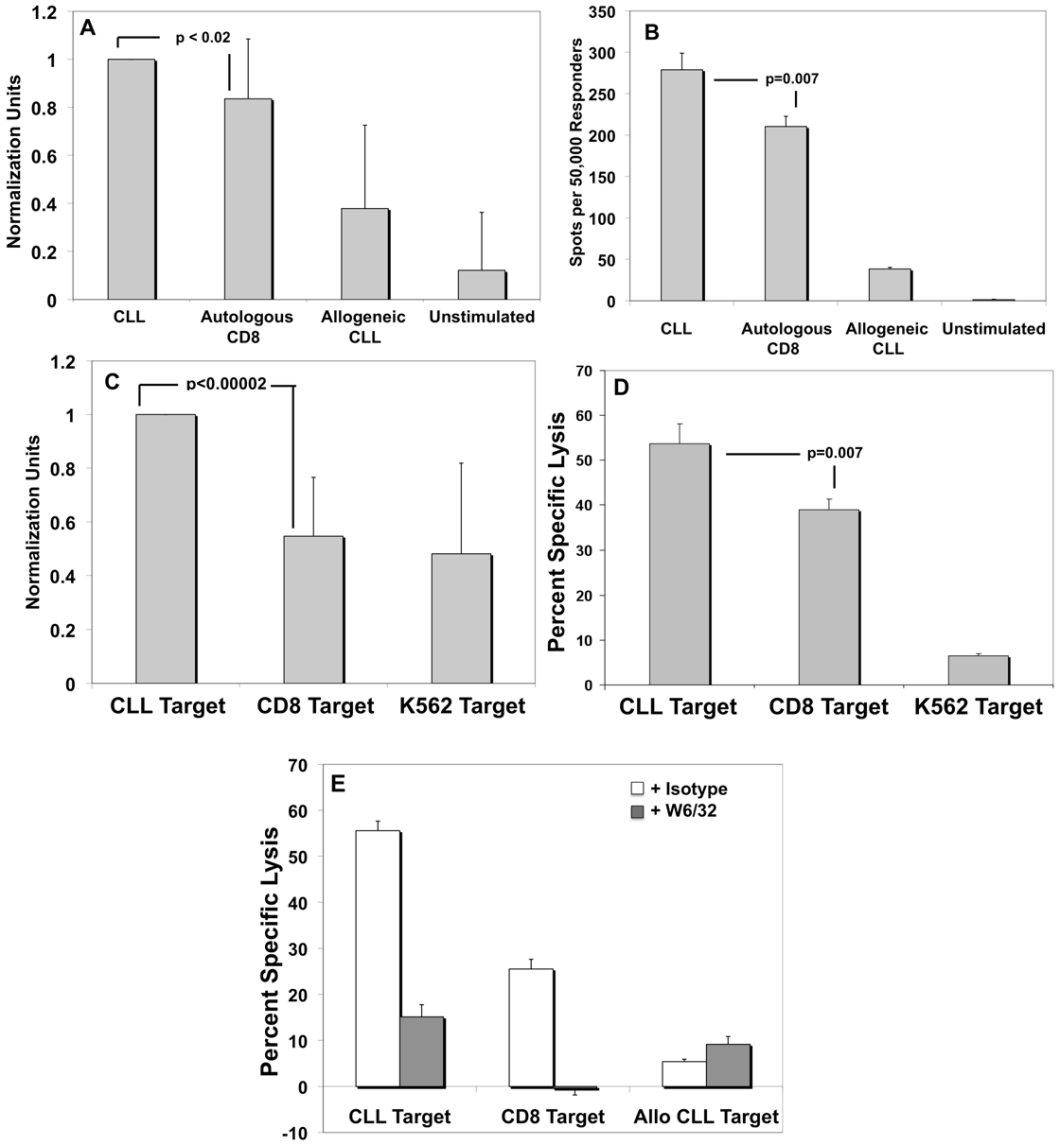
Partially HLA-matched UCB T-cells stimulated with CLL-APC preferentially react with and lyse the CLL cells against which they were primed in an MHC Class I-Dependent Fashion. A. Normalized data taken from nine independent experiments indicate UCB CLL-specific T-cells generated 20.3% more IFN-γ ELIspots (p = 0.02) when complexed with the UCB CLL cells against which they had been primed than when complexed with alloidentical stimulators. B. Raw data from representative experiment. C. These same cells could also mediate 79% more specific killing (p = 0.00002) when complexed with the CLL cells against which they had been primed than when complexed with alloidentical targets. D. Raw data from representative experiment. E. Lytic activity was abrogated by the W6/32 pan-MHC class I blocking antibody, demonstrating that lysis is mediated in an antigen-specific fashion through MHC class I/TCR interaction. White bars: lytic activity in the presence of control antibody. Gray bars: lytic activity in the presence of 1 µg/ml W6/32 antibody. Y-axis: percent specific lysis at E∶T ratio = 50∶1. For all five panels, error bars = +/−SD.

After two or three stimulations with CLL-APC, UCB T-cells were cultured with unmanipulated CLL cells to determine reactivity and specificity by IFN-γ ELISpot. To determine the specificity of the response to CLL antigens, normal CD3^+^ lymphocytes were isolated and expanded from the same patient that provided the CLL, and the CD4^+^ population was depleted to remove the possibility of allogeneic T regulatory cell (Treg)-mediated suppression of immune responses. Post expansion, we verified that the bead-activated CD3^+^ lymphocytes expressed MHC class II as do physiologically-activated human CD3^+^ cells (data not shown). Hence, the isolated CD3^+^CD4^−^ lymphocytes expressed the same class I and class II alloantigens as did the CLL cells and differences in spot number between CD3^+^ T-lineage control stimulators and CD19^+^ B-lineage CLL stimulators represent specificity against CLL or at least against B-lineage antigens absent from the T-lineage antigenic repertoire. We also analyzed levels of HLA-A/B/C expression on the surface of patient CLL and the corresponding CD8^+^ population. In no instances did CLL cells express higher levels of HLA-A/B/C than the corresponding CD8^+^ cells, validating the use of CD8^+^ cells as appropriate alloantigen controls (data not shown). Allogeneic CLL stimulators were also included as a control for NK cell activity. As demonstrated by the normalized composite of 11 different experiments (Figure 5A), CLL-primed UCB T-cells exhibited a significant increase in IFN-γ ELISpots when co-cultured with CLL stimulators in comparison to co-culture with HLA-identical CD8^+^ stimulators ***(average of all 9 experiments: 20.3%+/−21.2%, p<0.02 by pair-wise t-test***), indicating distinct, CLL–specific (or at least B-lineage specific) responses. Other controls such as CLL-only, CD8-only, and medium only were always devoid of spots and have been omitted for clarity. A representative experiment (1 of 11) is depicted in Figure 5B and the results of all 9 experiments may be found in Table 3. In the depicted representative experiment, CLL-primed UCB T-cells exhibited a 33% (p = 0.007) increase in IFN-γ ELISpots when co-cultured with CLL stimulators in comparison to co-culture with HLA-identical CD8^+^ stimulators.

**Table 3:** Summary of IFN-γ ELISpot Results.

Assay Number	IFN-γ ELISpots per 50,000 Total Cells			
	CLL Targets	CD8^+^ Targets	Allogeneic Targets	Unstimulated
1	198	140	N/A	78
2	63	83	N/A	42
3	18	8	N/A	0
4	279	210	38	1
5	280	236	31	1
6	287	318	137	1
7	362	262	341	2
8	404	338	92	2
9	276	218	N/A	1

### UCB T-cells stimulated by partially HLA-matched CLL-APC preferentially kill unligated CLL cells in an HLA-A/B/C restricted fashion

After three stimulations with CLL-APC, UCB effectors were co-cultured with ^51^Cr-labeled, unmanipulated CLL targets to determine killing ability by ^51^Cr release. Similar to the IFN-γ ELISpot assay, normal CD8^+^ lymphocytes autologous to the CLL APC were used as alloantigen controls. Labeled K562 cells served as controls for NK activity. ***Normalized data from 11 different experiments indicated that CTL killing of CLL targets was 79% higher (55.4% vs. 31.4% at E∶T = 20∶1) than that of the HLA-identical control targets (p = 0.00002 by pair-wise t-test,*** ***Figure 5C*** ***)*** **,** indicating a highly CLL–specific (or B-lineage specific) response. A representative experiment depicted in Figure 5D shows CLL-primed UCB T-cells lysing 54% of labeled CLL targets versus 39% of HLA-identical CD8^+^ targets, and the results of all 11 experiments may be found in Table 4. Though IFN-γ secretion was higher against CLL targets than control targets in 7 of 9 experiments, lytic killing of CLL targets was much higher than that of control targets in all 11 experiments. To verify that killing of targets was mediated by HLA class I/TCR interaction, the pan-HLA-A,B,C blocking antibody W6/32 was added to lytic assays. As demonstrated by the experiment in Figure 5E, addition of the W6/32 antibody was sufficient to reduce lysis of CLL targets by 73% (p = 0.00003) and lysis of the alloantigen CD8^+^ control by 100% (p = 0.00006). Lysis of allogeneic NK activity controls was unaffected by the addition of W6/32, in total indicating that lysis of allogeneic cells was due entirely to NK cells, that lysis of CD8^+^ targets was due entirely to T-cells, and that lysis of CLL targets was due predominantly to T-cell activity.

**Table 4:** Summary of ^51^Cr Lysis Results.

Assay Number	Target Lysis at E∶T = 20∶1		
	CLL Targets	CD8^+^ Targets	Allogeneic Targets (NK activity control)
1	54.9%	36.1%	50.4%
2	53.7%	39.0%	6.5%
3	61.4%	20.2%	43.6%
4	61.6%	36.9%	17.7%
5	55.2%	43.7%	3.5%
6	48.6%	7.0%	20.3%
7	51.9%	32.9%	19.7%
8	43.5%	25.0%	45.2%
9	42.8%	9.8%	28.9%
10	43.1%	23.9%	8.7%
11	92.2%	71.3%	N/A

### Adult-derived T-cells stimulated by partially HLA-matched CLL-APC cannot preferentially kill unligated CLL cells

**Figure 6:**
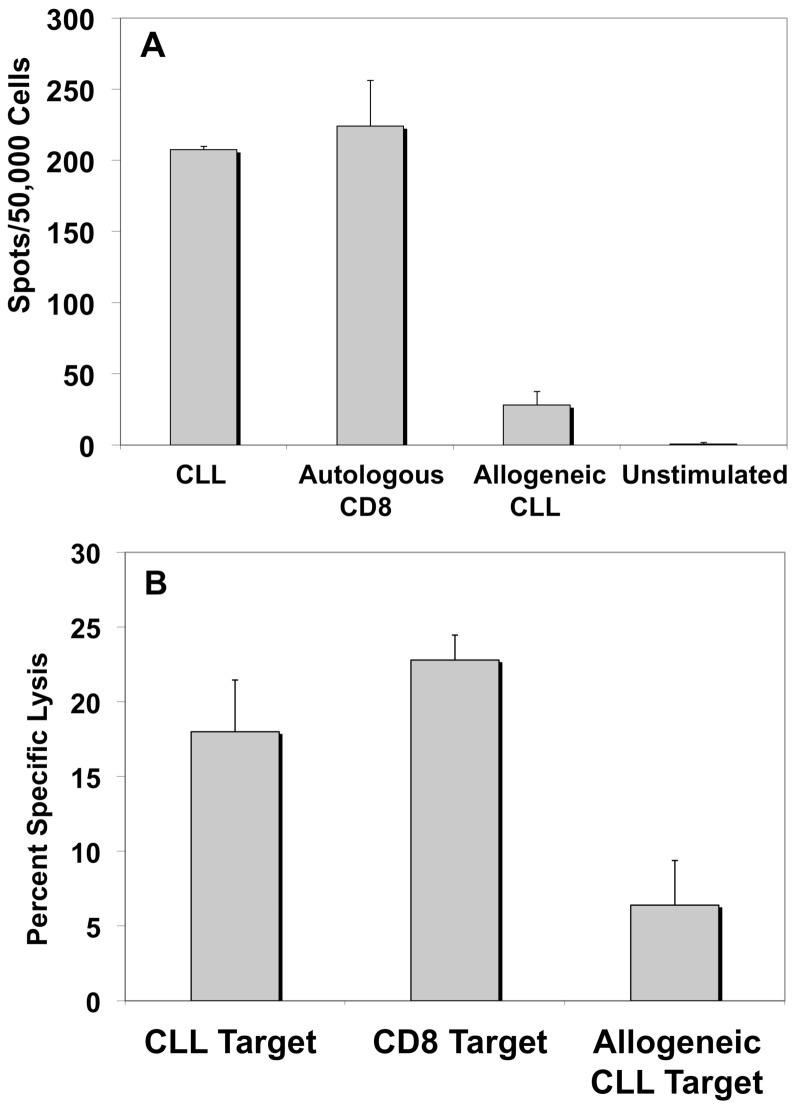
In contrast to UCB-derived T-cells, adult-derived T-cells do not demonstrate antigenic specificity against unligated CLL targets. A. IFN-γ ELISpot demonstrates no differences in immune reactivity between adult T-cells primed by CLL-APC and CD8^+^ targets or CLL targets. Y axis = IFN-γ ELISpots/50,000 cells. B. ^51^Cr killing assay shows adult T-cells primed by CLL-APC mediate higher levels of killing against the normal alloantigenic target (CD8^+^ cells) than against the unligated CLL target. Y axis = percent specific killing. For both panels, error bars = +/−SD.

Previous reports have indicated defects in the ability of adult (i.e. non-neonatal) peripheral blood-derived T-cells to interact immunologically with CLL targets in the absence of previous CD40 ligation. In other words, though the strategy of CD154 transfection and subsequent CD40 ligation has previously been used to generate CLL-APC and CLL-specific T-cells, such T-cells have typically been able to react only against CD154-transduced CLL targets ***but not against native, unmanipulated CLL targets*** [17]; [20]; [21]; [23]. In order to determine whether the use of cord blood T-cells genuinely imparts a distinct immunological advantage over the use of adult peripheral blood-derived T-cells, experiments were repeated using adult peripheral blood apheresis products matched at 4/6 HLA-A,B, or DR loci with patient CLL cells. Execution of this experiment was technically very challenging as there were relatively few normal donor apheresis products available, hence 4/6 HLA-matching to CLL patient cells was difficult to achieve. Ultimately, two different normal donor apheresis products were matched at 4/6 HLA loci to a single CLL patient. One of these normal donor products failed to expand at all when co-cultured with CD40-ligated CLL cells, a phenomenon that was not observed among 14 different co-culture experiments using UCB-derived T-cells. The second normal donor product expanded and was able to be analyzed. IFN-γ ELISpot indicated that adult-derived PBMCs could recognize alloantigen; however, there was no indication that CLL antigens were recognized with any kind of specificity (Figure 6A). Further, adult PBMC-derived T-cells primed by CLL-APCs were also unable to mitigate significant lysis of CLL cells, indeed, demonstrating a preferential lysis of the autologous CD8^+^ cells rather than the CLL cells against which they had been primed (Figure 6B). This lytic pattern was not observed among any of the experiments performed with partially-HLA matched UCB T-cells.

### Expanded UCB T-cells specific for alloantigens (GVH) can be separated from T-cells specific for leukemic antigens (GVL)

**Figure 7:**
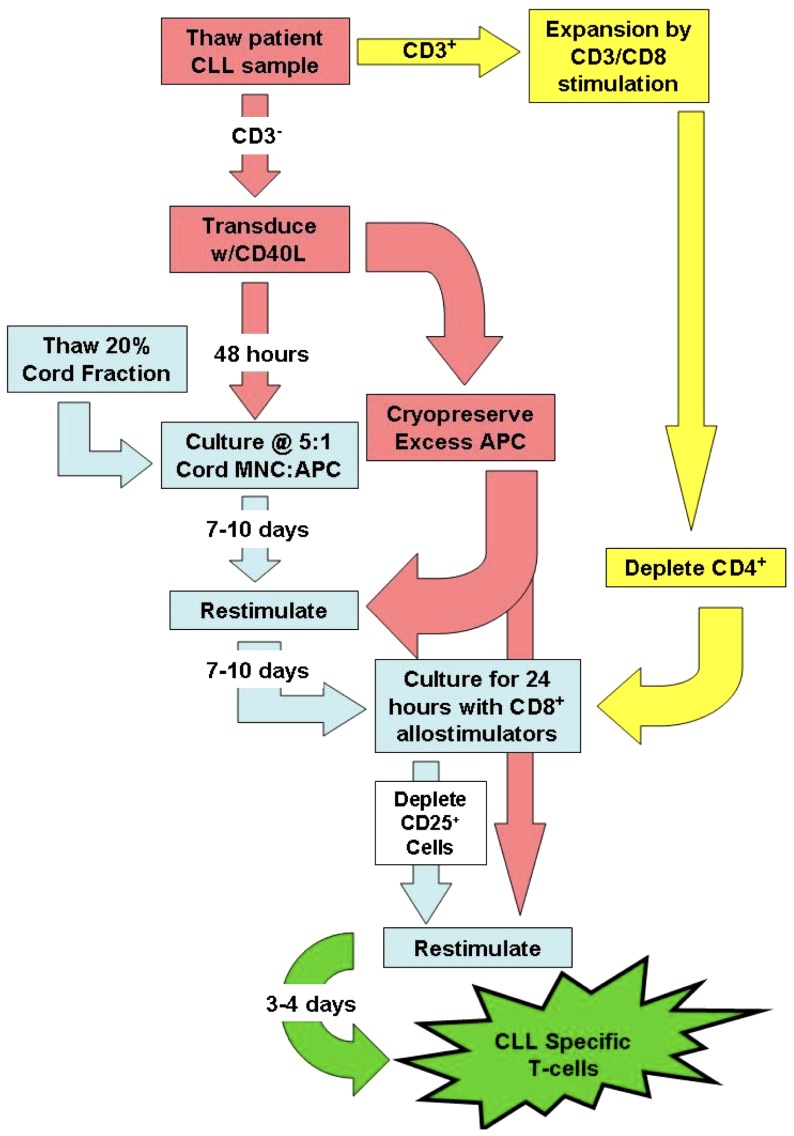
Flow chart schematic indicating the process of separating GVH from GVL during the generation of CLL-specific UCB T-cells.

**Figure 8:**
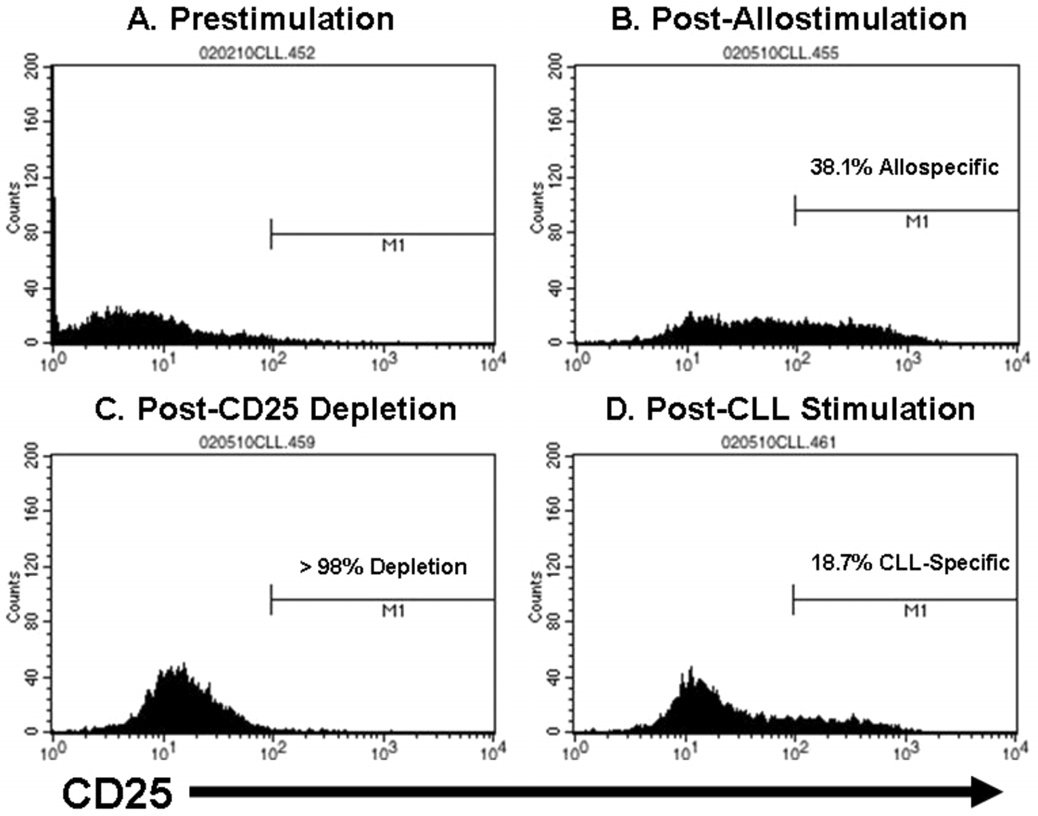
Separation of Alloreactive T-cell activity (GVH) and leukemia-specific T-cell activity (GVL). (A) CD25 expression of bulk T-cells prior to allostimulation. (B) CD25 expression of bulk T-cells post-allostimulation. (C) CD25 expression of allostimulated T-cells following depletion of CD25^+^ cells. (D) CD25 depleted cells following third stimulation with CLL-APC.

**Figure 9:**
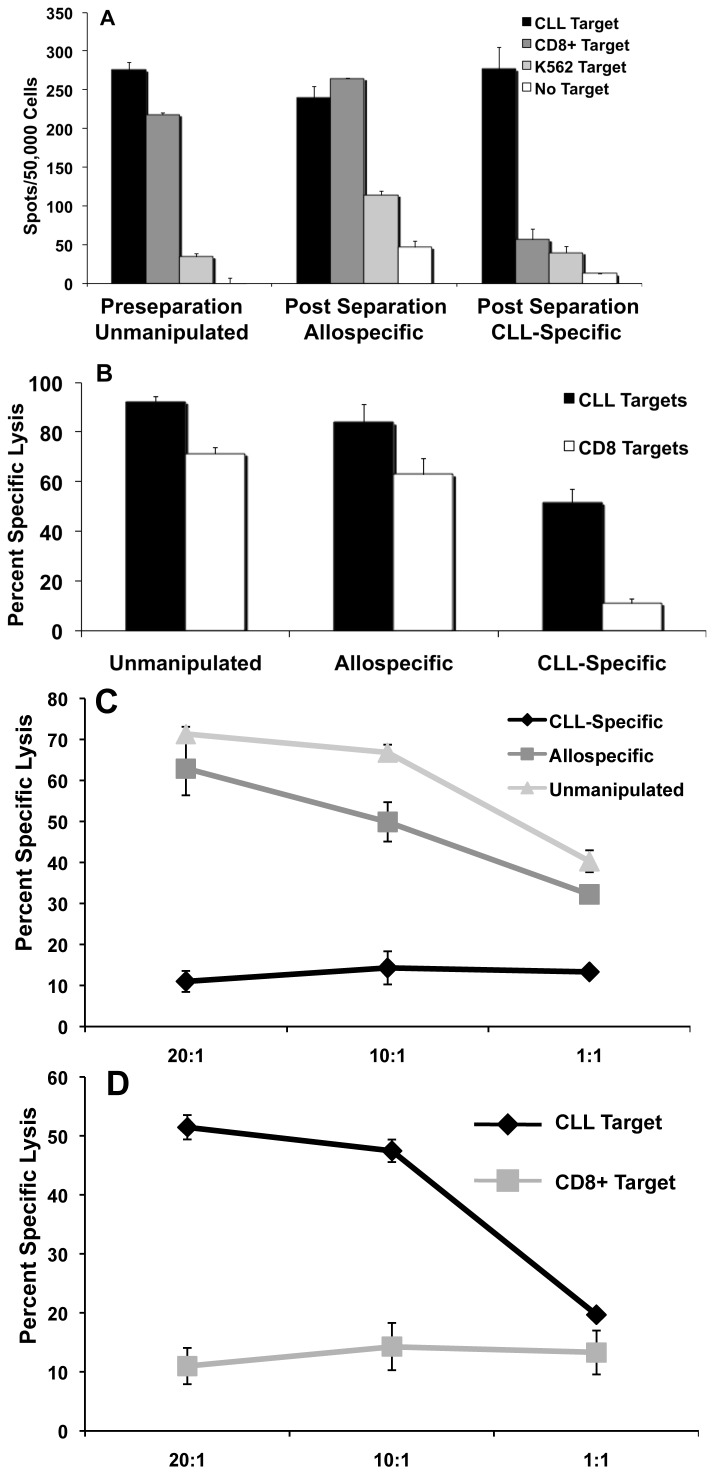
In Vitro Functional Analysis of Partitioned Leukemia-Specific T-cells. (A) IFN-γ ELISpot demonstrating the separation of alloreactive T-cell activity from leukemia-specific T-cell activity. Black bars – CLL stimulators. Dark gray bars – CD8^+^ stimulators. Light gray bars – K562 stimulators (control for NK cell activity). White bars – No stimulators. (B) ^51^Cr lytic assay demonstrating the separation of alloreactive T-cell activity from leukemia-specific T-cell activity. Black bars – CLL targets. White bars – CD8^+^ targets. X-axis: source of responder cells. E∶T ratio = 20∶1. C. Demonstration of this principle over a range of E∶T ratios, indicating that, while unmanipulated and depleted allospecific effectors lyse CD8^+^ alloidentical targets, the CLL-specific T-cells no longer demonstrate lytic specificity for the CD8^+^ targets. Light gray lines/triangles = unmanipulated effectors. Dark gray lines/squares = partitioned allospecific effectors. Black lines/diamonds = CLL-specific effectors. Targets consisted of non-leukemic but HLA-identical CD3^+^CD8^+^ cells. D. Conversely, the CLL-specific T-cells do not recognize alloidentical CD8^+^ targets but still recognize and lyse CLL targets with a high degree of specificity. Black lines/diamonds = CLL targets. Gray lines/squares = CD3+CD8+ targets. Effectors consisted of post-separation CLL-specific T-cells. For all four panels, error bars = +/− SD.

While the preceding data indicate that CLL-specific responses can be generated from partially-HLA matched UCB T-cells, the data also indicate that such responses are characterized by a high background of alloreactivity as would be expected when priming T-cells against an allogeneic stimulator. In order to render this approach appropriate for clinical application, we developed a GMP-compliant strategy for separating alloresponses (GVH) from leukemia-specific responses (GVL). Ten days after the second CLL-APC stimulation, bulk T-cells were stimulated with expanded, irradiated CD8^+^ cells derived from the same patient as was the leukemic clone. According to such a strategy, T-cells reactive against shared alloantigens (as well as shared B and T lineage antigens) respond and upregulate surface CD25 whereas T-cells reactive only against leukemic (or at least B lineage-specific) antigens remain CD25 negative. CD25^+^ T-cells (i.e. alloreactive cells) were then magnetically depleted after which CD25^−^ T-cells were stimulated a third time with CLL-APC. A schematic of this experimental design can be found in Figure 7. Figure 8 depicts the results of this separation strategy ***(one of three independent experiments).*** Figure 8A indicates few cells were CD25^+^ prior to stimulation with autologous CD8^+^ cells. Figure 8B then shows the significant fraction of cells that became CD25^+^ post allostimulation ***(range 34.1%–49.1%)***. Figure 8C shows the efficiency by which the CD25^+^ alloreactive cells can be depleted ***(range 95%–99%)***, and Figure 8D shows that the formerly CD25^−^ cells now upregulate CD25 in response to CLL-specific stimulation (range 11.5%–18.7%). Because the CLL stimulators and the CD8^+^ stimulators are alloidentical, the T-cells that could not upregulate CD25 following CD8^+^ cell stimulation but do upregulate CD25 following CLL cell stimulation must have a specificity for CLL (or at least B-linage) antigens not expressed or presented by the CD8^+^ population. Figure 9A shows a representative ELISpot experiment ***(one of three)*** in which the percentage of alloreactive cells is reduced from 76.0% (pre-separation unmanipulated) to 6.8% (post-separation CLL-specific), with no CLL-specificity retained among the depleted cells (post-separation allospecific). Figure 9B shows a representative CTL assay ***(one of three)*** at E∶T = 20∶1 in which lysis of autologous CD8^+^ targets by pre-separation CTL was 71.3% (versus 92.2% lysis of CLL targets) whereas CD8^+^ lysis by CLL-specific CTL was only 11.0% (versus 51.5% of CLL targets). Figure 9C demonstrates this principle over a range of E∶T ratios, demonstrating that, while unmanipulated and depleted allospecific effectors lyse CD8^+^ alloidentical targets, the CLL-specific T-cells no longer demonstrate lytic specificity for the CD8^+^ targets. Conversely, Figure 9D indicates that the CLL-specific T-cells, while they do not recognize CD8^+^ targets, still recognize and lyse CLL targets with a high degree of specificity.

### UCB T-cells stimulated by partially HLA-matched CLL-APC control CLL *in vivo* and do not cause xenoGVHD

**Figure 10:**
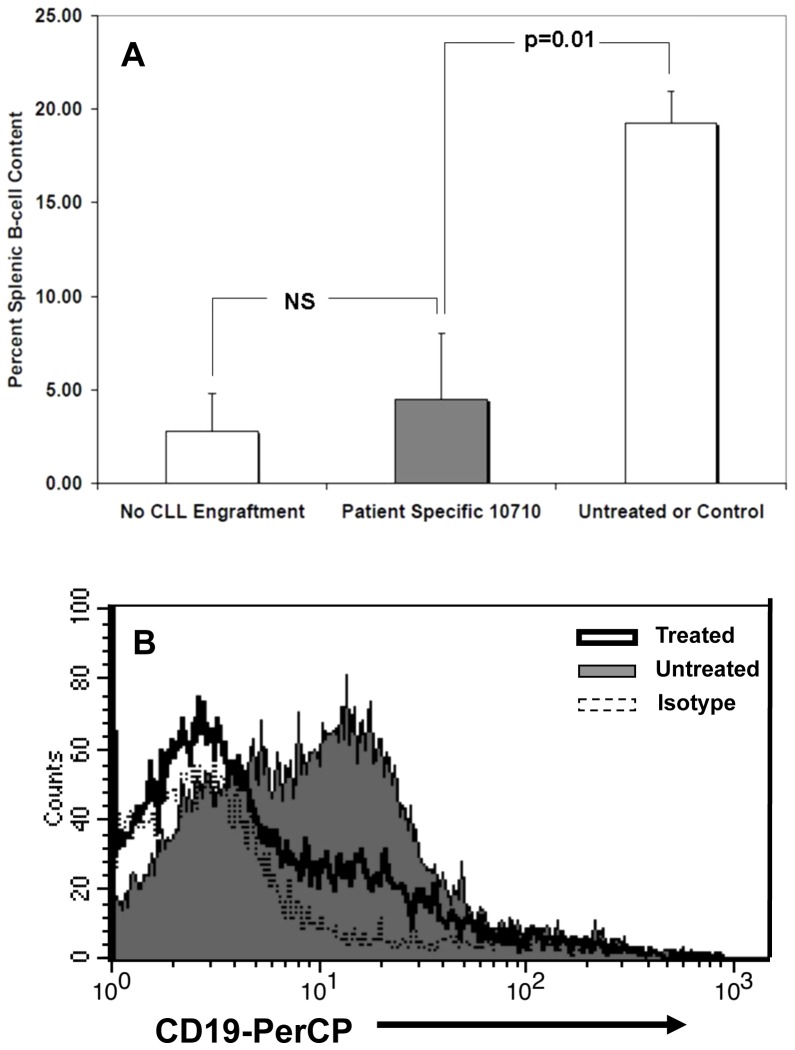
In Vivo Functional Analysis of Partitioned Leukemia-Specific T-cells. A. Administration of CLL-specific T-cells *in vivo* to CLL-engrafted mice resulted in a reduction of splenic B-cell burden from 19.3% in untreated or control-treated mice to 4.5% (p = 0.01) among mice treated with CLL-specific T-cells This was statistically indistinguishable from the 2.8% splenic B-cell content of non-CLL engrafted mice. B. Representative flow cytometry plot of splenic CD19^+^ cells. Dashed line/unfilled = isotype control. Thick black line/unfilled = CLL engrafted and treated with CLL-specific effectors. Thin black line/gray filled = CLL engrafted but untreated.

To demonstrate the ability of CLL-specific T-cells to function appropriately *in vivo* without concomitant development of xenoGVHD, we engrafted immunodeficient mice with a CLL clone [29] against which CLL-specific T-cells had previously been generated. Following engraftment, cohorts of 4 animals each were treated with the 10710 CLL-specific CTL line (5×10^6^ per mouse), while others were left untreated or treated with CTL lines generated against a different leukemic clone (referred to as non-specific CTLs). Six weeks later, mice were sacrificed and splenic B-cell content examined by flow cytometry. As depicted by Figures 10A and 10B, untreated or control treated mice exhibited a splenic B-cell content of 19.3+/−1.7% whereas mice treated with CLL-specific T-cells exhibited a splenic B-cell content of 4.5+/−3.5% (p = 0.01). The 4.5% splenic B-cell content of treated mice was not statistically distinct from a cohort of mice (2.8+/−2.0%) that was not engrafted with CLL. At the end of the experiment all the treated mice were healthy, exhibiting no histological characteristics or behaviors associated with xenoGVHD (data not shown) within a time frame more than sufficient for GVHD to develop [30].

## Discussion

Recent work has demonstrated the vital role that both allospecific and CLL antigen-specific T-cells play in the generation of durable remission following nonmyeloablative HSCT [31]. Yet the development of such effectors for active, specific immunotherapy of CLL might be significantly compromised by the ability of CLL cells to efficiently suppress T-cell mediated immune responses [20]–[22]. Ramsay et al [23] have suggested that T-cells derived from UCB grafts might be less susceptible to the immunosuppressive effects of CLL cells, results that provided the impetus for the present work. Here we demonstrate that functional, CLL-specific T-cells can be generated by priming UCB T-cells with partially HLA-matched, CD40-ligated CLL cells. Importantly, these CLL-specific T-cells retain CTL function when cultured with unmodified CLL targets. Functionality was demonstrated by immune synapse assay, multiparametric intracellular flow cytometry, IFN-γ ELISpot, ^51^Cr release, and suppression of CLL disease in a humanized *in vivo* mouse model. Though functional comparison of UCB-derived T-cells with adult PBMC-derived T-cells was technically challenging due to the difficulty in finding sufficiently HLA-matched normal donor products, the comparison performed did support the hypothesis that UCB-derived T-cells possess an immunological advantage allowing mitigation of CLL-mediated immunosuppression. In this experiment, ELISpot data demonstrated that CD40-ligated CLL-APC were unable to generate adult donor-derived T-cells with any antigenic specificity, other than that against alloantigens. Further, ^51^Cr lytic data demonstrated that adult donor-derived PBMC were unable to lyse CLL targets with the same efficiency as their UCB-derived counterparts. While the ability of UCB-derived T-cells to circumvent CLL-mediated immune suppression is significant, the mechanism of circumvention is not understood. The most obvious difference between UCB-derived T-cells and APB-derived T-cells is that of the memory phenotype. Whereas T-cells from the UCB cell product are almost entirely naïve, T-cells in peripheral circulation are predominantly of an effector memory phenotype. Unpublished work from our laboratory has indicated that the subcellular localization of the Src-family kinase p56/Lck is significantly perturbed in NK cells following direct contact with CLL cells, resulting in the downregulation of critical signaling events at the immune synapse. Interestingly, a similar method of immune suppression is employed by the HIV virulence factor nef, the expression of which directs the localization of Lck to extrasynaptic sites within HIV-infected CD4^+^ lymphocytes [32]–[34]. Data from the literature suggest that expression of Lck is relatively low in UCB T-cells [35] and that, in contrast to memory T-cells, Lck localization in naïve T-cells is predominantly cytoplasmic [36]. Given these data, we would hypothesize that differential patterns of Lck expression and localization in naïve, UCB T-cells could account for the relative insensitivity of these cells to a major CLL mechanism of immunosuppression. Nevertheless, this hypothesis has yet to be formally investigated, and in the absence of definitive mechanistic insight, the conclusion that UCB T-cells are less susceptible to CLL-mediated immune suppression than adult PBMC-derived T-cells necessarily remains a preliminary finding.

The immunosuppressive effects of CLL upon T-cells are well-documented in the literature [17]; [20]; [21]; [23]; however, there is at least one report suggesting good immune recognition of CLL by adult-derived PBMCs following co-culture with CD40-ligated CLL-APC [37]. In this report, the authors generated CLL-APC by incubation of primary CLL in 500 U/ml IL-4 in conjunction with CD40 ligation, generating CLL-reactive CTL from CD4-depleted bulk T-cells. In some cases, multiply-stimulated CTLs appeared to lyse primary CLL with high efficiency; however, the CTLs almost always lysed CD40-ligated CLL with a higher efficiency, the authors provided no alloantigen control by which to determine the presence of CLL-specific responses, and no comparison to cord blood was provided. Given these important variables, it is difficult to compare these data to the current work in a meaningful fashion.

Though we demonstrate lineage-specific immune recognition above and beyond the background of alloantigenicity, the background of allospecific responses is necessarily high when UCB grafts are HLA-matched to the CLL patient product at only 4 of 6 HLA loci. The level of alloreactivity observed among the unmanipulated CTL was clinically unacceptable, necessitating a strategic modification of the protocol. We developed a GMP-compliant protocol that could effectively separate alloreactive CTL (GVH) from leukemia-specific CTL (GVL). This strategy involved the stimulation of bulk CTL with non-leukemic cells HLA-identical to the leukemic clone (i.e. patient normal cells) followed by a CD25 magnetic depletion with a GMP-compliant reagent. The remaining CTL were shown to be specific for the B-lineage leukemic clone and non-reactive against the T-lineage cells that served as stimulators for the CD25 depletion. Somewhat related strategies have been described in the literature [38]–[40], most notably by Samarasinghe et al [40]. In this recent publication, the authors used EBV-transformed LCLs to stimulate alloreactive cells among HLA-mismatched normal donor products. There are several reasons why the use of bead-expanded, irradiated CD8 cells might be preferable to the use of EBV-transformed LCLs. Some of these reasons are technical (i.e. the relative difficulty of generating LCL lines for every patient); some are related to patient safety (i.e. issues encountered by the introduction of EBV in the immunocompromised setting); and some are regulatory (i.e. bead expansion of patient T-cells is already part of an FDA approved clinical trial currently underway at the MD Anderson Cancer Center). Further, Samarasinghe did not demonstrate that their LCL-depleted T-cell product possessed reactivity against primary leukemia [40] as we do here. More practically, B-cell derived LCLs would not be appropriate for use as stimulators in the setting of CLL since LCLs potentially share important antigens with the B-lineage leukemic clone.

Recently, Porter et al described the successful application of chimeric antigen receptor (CAR) technology to treat refractory CLL [41]. In this study, patient T-cells were transduced with a CAR construct recognizing the B-cell CD19 surface antigen and then reinfused following *ex vivo* expansion. This exciting development has reinvigorated the entire field and has engendered cautious optimism that this and other cell therapy treatment regimens might one day prove to be viable therapeutic options for CLL and other important malignancies. It is worth noting, however, that many other investigators previously failed to produce similar results using essentially identical systems as that used by Porter. Further, the reported results were of a very small cohort for which a successful response was observed in 3 of only 4 treated patients. Given these caveats, we feel the translatability of this approach on a large scale remains to be definitively demonstrated. Additionally, the system described here offers a significant advantage over CAR therapy in that that UCB T-cells (which are eventually given to the recipient) do not have to undergo transfection and potential genetic perturbation for eventual tumor-specific functionality.

It would have been ideal to demonstrate in vitro separation of GVL from GVH by using a panel of different cell types derived from the patient. Unfortunately, this was not technically feasible. Most patient leukodepletion products consisted almost entirely of CLL cells, and it was only possible to generate substantial number of autologous control CD8^+^ cells by the isolation and expansion of very rare CD3^+^ cells. There is no analogous expansion protocol for other cell types, nor can significant numbers of normal B-cells typically be readily discerned from the neoplastic B-CLL population. Further, most of the patient products released for experimentation were derived from individuals whom were already deceased or whom had been lost to follow-up, rendering impossible or impractical the collection of cells from patients in remission or the generation of autologous cell lines from patient tissues. Though definitive conclusion that GVH and GVL have been separated will require prospective validation of this strategy using a panel of autologous hematopoietic cells isolated from patients in remission, the absence of xenoGVHD in an animal model supports this hypothesis. The analogy is not perfect given that the murine alloantigens were not identical to the CLL alloantigens that reacted non-specifically with the UCB graft T-cells. Nonetheless, because of the significant cross-reactivity that exists between human and mouse MHC, the experiment demonstrates a highly focused specificity of response *in vivo* such that all T-cell alloreactivity with the MHC class I H-2^g7^ murine haplotype was lost.

Importantly, the depletion strategy described here might be modified so as to be applicable not only to adoptive therapy, but to a variety of other settings as well. The strategy is dependent solely upon pretreatment of the therapeutic product with patient-derived allostimulators and the ability to remove CD25^+^ cells in a GMP-compliant fashion. The generation of allostimulators is straightforwardly accomplished by CD3/CD28 stimulation of patient T-cells, and efficient removal of CD25^+^ cells is accomplished by the use of magnetic separation technologies, two techniques that are well within the routine capabilities of most academic cell therapy centers. Hence this separation strategy, in one of several different forms, might ultimately be useful in the setting of allogeneic hematopoietic stem cell transplantation, solid organ transplantation, and regenerative medicine applications.

In summary, we describe a novel strategy by which UCB-derived, CLL-specific T-cells might be generated for therapeutic applications. Despite the ability of CLL to induce T-cell immunosuppression, UCB-derived T-cells appear to retain functionality when co-cultured and assayed with unmanipulated CLL targets. T-cell specificity encompassed both alloantigens and CLL-specific antigens; however, alloreactive T-cells were tentatively separated from leukemia-specific T-cells by means of a straightforward, GMP-compliant strategy. The tentative ability to separate alloreactive T-cells (GVH) from leukemia-specific T-cells (GVL) *in vitro* is a crucial component of this particular strategy but might also be applicable to other settings as well. T-cell allotherapy for patients with CLL as indicated by our results could be provided under several clinical scenarios including 1) as a singular cellular therapy in the setting of lymphodepleting chemotherapy [42]; 2) as a third party source of tumor reactive T-cells in the setting of traditional, matched-related or matched-unrelated HSCT; or 3) as an adjunct to double UCB HSCT. In the latter scenario, one could conceive of expanding a fraction of a single UCB unit to generate tumor-specific T cells and administering as a DLI in the peri-HSCT, minimal residual disease setting. The data support the further development of this novel procedure as a therapeutic modality for CLL in conjunction with UCBT as a means of preventing or treating relapsed disease.
